# A Novel Nonsense Mutation in *PANK2* Gene in Two Patients with Pantothenate Kinase-Associated Neurodegeneration

**Published:** 2016-10-23

**Authors:** Soudeh Ghafouri-Fard, Vahid Reza Yassaee, Alireza Rezayi, Feyzollah Hashemi-Gorji, Nasrin Alipour, Mohammad Miryounesi

**Affiliations:** 1 *Department of Medical Genetics, Shahid Beheshti University of Medical sciences, Tehran, Iran.*; 2 *Genomic Research Center, Shahid Beheshti University of Medical Sciences, Tehran, Iran.*; 3 *Pediatric Neurology Department, Loghman Hospital, Faculty of Medicine, Shahid Beheshti University of Medical Sciences, Tehran, Iran.*

**Keywords:** *PANK2*, pantothenate kinase-associated neurodegeneration, mutation

## Abstract

Pantothenate kinase- associated neurodegeneration (PKAN) syndrome is a rare autosomal recessive disorder characterized by progressive extrapyramidal dysfunction and iron accumulation in the brain and axonal spheroids in the central nervous system. It has been shown that the disorder is caused by mutations in *PANK2* gene which codes for a mitochondrial enzyme participating in coenzyme A biosynthesis. Here we report two cases of classic PKAN syndrome with early onset of neurodegenerative disorder. Mutational analysis has revealed that both are homozygous for a novel nonsense mutation in *PANK2* gene (c.T936A (p.C312X)). The high prevalence of consanguineous marriages in Iran raises the likelihood of occurrence of autosomal recessive disorders such as PKAN and necessitates proper premarital genetic counseling. Further research is needed to provide the data on the prevalence of PKAN and identification of common *PANK2* mutations in Iranian population.

Pantothenate kinase-associated neurodegen-eration (PKAN) syndrome is a rare autosomal recessive disorder characterized by progressive extrapyramidal dysfunction and iron accumulation in the brain and axonal spheroids in the central nervous system. Its prevalence has been estimated to be one to three in 1,000,000 (1). Brain magnetic resonance imaging (MRI) usually shows the ‘eye of the tiger’ pattern in the globus pallidus on T2 weighted which is due to iron deposition in the periphery (hypointensity) and necrosis on its central part (hyperintensity) (2). Dystonia, dysarthria, and dysphasia are the main clinical presentations. At later stages of disease course, other signs such as dementia, severe mental retardation and severe movement disability may occur (3). Other rare clinical manifestations are rigidity, parkinsonism, choreoathetosis, seizures, optic atrophy, and pigmentary retinopathy (2). In the classic form of PKAN, clinical features are detected in the first decade of life, while atypical form has a slower progression with clinical manifestations appearing in the second decade (4). Pantothenate kinase 2 (*PANK2*) gene located on 20p13 is the only gene whose mutations have been detected in PKAN syndrome (5). This gene has 34761 bps and 8 exons. The size of the longest transcript variant is 8649. Clinically, PKAN shares all the hallmarks of a mitochondrial disorder which is consistent with the PANK2 localization in the mitochondria and its role as the principle regulatory enzyme in coenzyme A biosynthesis (6). Coenzyme A participates in several metabolic pathways, such as the citric acid cycle, sterol and steroid biosynthesis, heme biosynthesis, amino acid synthesis, and β-oxidation. Depletion of coenzyme A due to *PANK2* mutations leads to diverse metabolic defects such as reduced lipid and cholesterol biosynthesis and defects in bile acid conjugation. Although PKAN patients have not been previously reported to have mitochondrial respiratory chain deficiency, some reports have demonstrated the elevation of lactate in these patients which is suggestive of possible mitochondrial dysfunction (7).

## Case Report

**Fig. 1:**
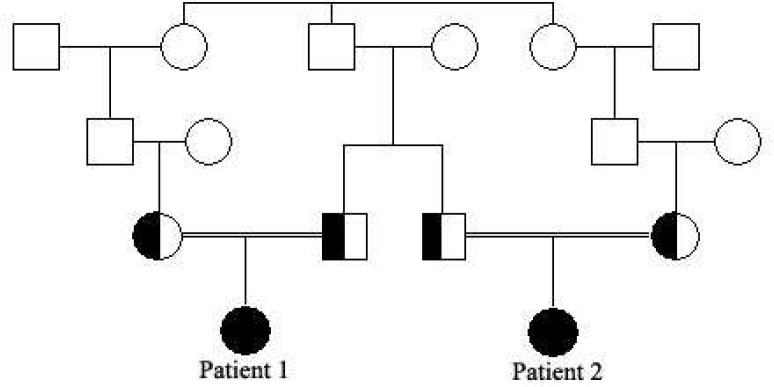
The pedigree of family showing the segregation data of avaialable cases

The first patient was an 11-year-old girl, the first child of healthy consanguineous Iranian parents (Figure 1). She was delivered by an elective cesarean section after an unremarkable pregnancy. The first signs and symptoms of disorder have been detected in the second year of life after she was noticed because of unsteady gait and frequent falls. Mild developmental delay had been noticed afterwards. Her cognition progressively worsened and she lost expressive speech at the age of 6. On physical exam, she had dystonia, dysarthria, dysphasia, spasticity and hyperreflexia. She was unable to walk without aid. Serum biochemistry test including serum ferritin, albumin, ceruloplasmin, copper, creatine kinase and liver function test were normal. Furthermore, urine organic acid panel was normal. Brain MRI showed decreased signal intensity of globus pallidus on either side. Magnetic resonance spectroscopy revealed signal void abnormality in globus pallidus compatible with spectrum of iron deposition disorders.

The second case was her 22 month old cousin which had been referred to genetic counseling because of her developmental delay and inability to walk. Due to parents’ anxiety, brain MRI has been done and revealed no abnormality.

**Fig. 2:**
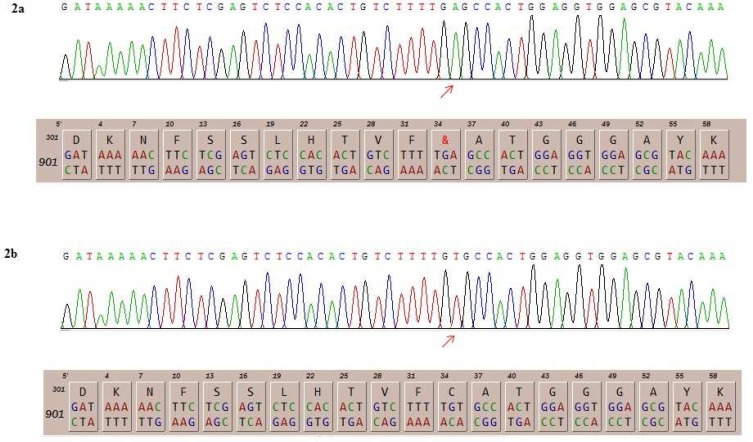
The detected mutations in patients and its consequence on the amino acid sequence (2a) and the normal DNA and protein sequence of the corresponding segment (2b).

Blood samples were collected from patients in EDTA tubes. Informed consents were obtained from parents before participation in the study in accordance with the protocol approved by local institutional ethics committee. DNA was isolated using the standard salting out method. Whole exome sequencing was performed using Illumina’s genome analyzer for the first patient with focus on 2752 OMIM disease genes (BGI-Clinical Laboratories, Shenzhen, China). The result showed the novel nonsense mutation c.T936A (p.C312X) in exon 2 of *PANK2* gene which was consistent with the diagnosis of PKAN. Afterwards, the 575 bp fragment corresponding to exon 2 of *PANK2* gene was amplified in both patients using the primer pairs 5'-TTGAAATAAGTTGCTACTGTGG-3 and 5'-CACTAGCGTACCTTTAATCTTC-3'. For this reason, 100 ng of genomic DNA was used in a total volume of 25 µl reaction mixture by Taq DNA Polymerase Master Mix Red (Ampliqon, Denmark). The PCR conditions were as follows: denaturation at 94°C for 4 min, then 30 cycles of denaturation at 94 °C for 30 s, annealing at 55.3 °C, and extension at 72 °C for 60 s, apart from the final cycle, for which extension was for 4 min and 30 s. Sanger sequencing of this segment was performed using the ABI Prism 3130 Genetic Analyzer (Applied Biosystems, Foster City, CA, USA) which confirmed the presence of homozygous c.T936A mutation in both patients (Figure 2). Targeted sequencing on the parents demonstrated that both were heterozygous for the identified variant. This variant has not been reported in generalist polymorphism databases (ExaC or exome variant server (EVS)), dbSNP and 1000 genome project. 

## Discussion

Clinical picture of the first patient was consistent with classic PKAN based on her clinical features (dystonia, rigidity, dysarthria, dysphagia, and gait disturbance) which started in the second year of life and progressed rapidly afterwards. In addition, brain MRI showed the typical signs associated with PKAN. Mutational analysis confirmed the diagnosis as well. Up to now, more than 100 mutations have been reported in *PANK2* in PKAN patients in different populations (8). The detection of *PANK2* mutation facilitates accurate diagnosis and permits presymptomatic testing of family members (9). Previous reports have demonstrated that in patients with two loss-of-function alleles, symptoms are always presented at an early stage of life. However, in the presence of missense mutations, it has been claimed that residual activity of the PANK2 determines the age of onset but not the progression of disorder (3). Previously, few reports have demonstrated *PANK2* mutations in Iranian patients (Table 1). The detected nonsense mutation in the present study has not been reported previously. The data presented in Table 1 suggest that despite the overall rarity of PANK, the mutation spectrum in Iranian population might be wide reducing the possibility of existence of a founder effect in this population. In addition, a certain mutation can be associated with both classic and atypical forms of the disorder in different families which can be attributed to gene interactions with other genetic as well as environmental factors. Such phenotypic diversity complicates identifi-cation of genotype-phenotype correlations which is a fundamental step in genetic counseling and prenatal diagnosis. The high prevalence of consanguineous marriages in Iran raises the likelihood of occurrence of autosomal recessive disorders such as PKAN and necessitates proper premarital genetic counseling. Further research is needed to provide data on the prevalence of PKAN and identification of common *PANK2* mutations in Iranian population.

**Table 1:** Reported *PANK2* mutations in Iranian patients

**Mutation**	**Zygosity**	**Number of patients**	**Clinical manifestations**	**Reference**
c.C1069T (p.Arg357Trp)	Homo	Three siblings/ One unrelated patient	Atypical form/ Classic form	(2)/(10)
c.1017-1020delAGAT insGCTTTGCAAAC	Homo	One patient	Classic form	(11)
c.G1442C (p.Arg481Pro)	Homo	One patient	Classic form	(10)
c.C1594T (p.Arg532Trp)	Homo	One patient	Classic form	
c.A1168T (p.Ile390Phe)	Homo	One patient	Classic form	
c.G833T (p.Arg278Leu)	Homo	One patient	Classic form	
c.A1208T (p.Asp403Val)	Homo	One patient	Atypical form	
c.A700C (p.Thr234Pro)	Homo	One patient	Atypical form	
c.T936A (p.Cys312X)	Homo	Two related patients	Classic form	Present study

## Conflicts of Interest:

The authors declared no conflict of interest.
